# Prediction of hypokalemia in patients with ST-segment elevation myocardial infarction: development and validation of a model with prehospital applicability

**DOI:** 10.3389/fcvm.2026.1776702

**Published:** 2026-03-27

**Authors:** Qian Gu, Chao Tang, Baojun Yang, Fei Shi, Xiaosong Gu, Jing Zhu

**Affiliations:** 1 Department of Cardiology, The Second Affiliated Hospital of Soochow University, Suzhou, China; 2 Department of Emergency, Suzhou Dushu Lake Hospital, Suzhou, China

**Keywords:** hypokalemia, nomogram, prediction model, pre-hospital assessment, ST-segment elevation myocardial infarction

## Abstract

**Background:**

Hypokalemia is common in patients with ST-segment elevation myocardial infarction (STEMI) and significantly elevates the risk of life-threatening arrhythmias and mortality. Yet no validated prehospital prediction tool exists to identify this high-risk condition early.

**Objective:**

To develop and validate a prediction model for hypokalemia in STEMI patients using readily available clinical and electrocardiographic parameters that are fully accessible in prehospital settings, and to systematically evaluate its prehospital application potential.

**Methods:**

A retrospective observational study was conducted involving 320 STEMI patients admitted to the Second Affiliated Hospital of Soochow University between January 2023 and December 2024. Patients were categorized into hypokalemia (*n* = 114) and non-hypokalemia (*n* = 206) groups based on initial serum potassium levels. Univariate logistic regression, least absolute shrinkage and selection operator (LASSO), and multivariate logistic regression were used to identify independent predictors. A nomogram was constructed and evaluated for discrimination, calibration, and clinical utility.

**Results:**

Five independent predictors were identified: symptom-to-door time (OR = 0.85, 95% CI: 0.78–0.94), syncope/coma (OR = 3.57, 95% CI: 1.12–11.37), atrial arrhythmia (OR = 4.18, 95% CI: 1.33–13.17), PR interval (OR = 1.01, 95% CI: 1.00–1.02), and U wave (OR = 5.20, 95% CI: 2.59–10.46). The prediction model demonstrated good discrimination with an AUC of 0.735 (95% CI: 0.680–0.791). Calibration curves and decision curve analysis confirmed satisfactory model performance and clinical usefulness.

**Conclusion:**

We developed and validated a practical nomogram for predicting hypokalemia risk in STEMI patients using five variables readily available in prehospital and emergency settings. This tool enables early risk stratification, facilitates targeted intervention in high-risk individuals, and guides early potassium supplementation. It may improve prehospital care and clinical outcomes in STEMI patients.

## Introduction

Heart function fundamentally depends on the precise generation and propagation of action potentials, in which potassium channels play a crucial role. Hypokalemia is defined as a serum potassium level below 3.5 mmol/L, which severely disrupts the stability of cardiac electrophysiology and leads to the occurrence of arrhythmia, especially during acute myocardial ischemia (1). After myocardial infarction, fibroblasts establish electrical coupling with surviving myocardial cells, causing abnormal changes in potassium channels and inducing arrhythmia (2). Multiple clinical studies have also confirmed that abnormal potassium ion concentration significantly increases the risk of malignant arrhythmia and affects patient prognosis (3, 4). The risk of arrhythmia can be significantly heightened in MI patients with hypokalemia even before revascularization is performed (5).

Hypokalemia patient may present with symptoms such as weakness, nausea, vomiting, coma, or syncope—manifestations that can overlap with those of myocardial infarction. In clinical practice, it is not accurate to identify concurrent hypokalemia in ST-segment elevation myocardial infarction (STEMI) patients based solely on single symptoms or physical signs. The diagnosis of hypokalemia often relies on the detection of venous potassium levels. The unique susceptibility of cardiomyocytes to extensive damage from even brief ischemia means that patients may already be in the catheterization lab for revascularization before their hypokalemia is even identified. The electrophysiological instability induced by PCI and ischemia-reperfusion injury, aggravated by hypokalemia, results in a markedly increased susceptibility to and higher incidence of arrhythmias (6).

Although there have been studies exploring the relationship between blood potassium levels and STEMI prognosis, most research has focused on in-hospital blood potassium monitoring, and there is relatively little research on predicting and intervening in pre-hospital blood potassium levels. If the high-risk population for hypokalemia can be quickly identified before or in the early stages of admission, and targeted interventions (such as preventive potassium supplementation) can be implemented, it may effectively reduce the incidence of malignant arrhythmias and improve patient prognosis. However, there is currently a lack of multi-indicator combination hypokalemia prediction models suitable for pre-hospital environments.

Therefore, this study aims to retrospectively analyze the emergency admission baseline data of STEMI patients, explore independent risk factors for early hypokalemia after STEMI onset, construct and validate a hypokalemia risk prediction model using variables fully accessible in prehospital emergency scenarios, and clarify its potential value in prehospital clinical decision-making. Notably, all variables to be included in this study were preset to be obtainable synchronously through prehospital inquiry, physical examination and 12-lead electrocardiogram, without relying on laboratory test results, which lays a core foundation for the translation and application of the model in prehospital scenarios.

## Materials and methods

A retrospective observational study was conducted to collect cases of STEMI patients who were admitted to the emergency room of the Second Affiliated Hospital of Soochow University from January 2023 to December 2024. Inclusion criteria comprised: (1) The diagnosis of patients with STEMI meets the diagnostic criteria of the 4th edition of the Global definition of myocardial infarction (2018) (7); (2) Age ≥ 18 years, electrocardiogram-confirmed STEMI, and primary percutaneous coronary intervention with stent deployment within 12 h of symptom onset; (3) Intravenous blood sampling for serum potassium completed within 1 h of emergency department arrival; (4) Capable of communication, without psychiatric disorder, and able to provide accurate and valid clinical information. Exclusion criteria were as follows: (1) Patients with incomplete clinical data (e.g., missing ECG or serum potassium records); (2) Presence of atrial fibrillation, atrial flutter, or any atrioventricular block on the emergency ECG, precluding reliable interpretation of PR intervals and *P* waves; (3) Administration of potassium supplementation or medications significantly altering potassium homeostasis before hospital admission; (4) Receipt of life-sustaining measures (e.g., endotracheal intubation, extracorporeal membrane oxygenation) prior to blood draw, or patients who died prior to hospital arrival; (5) End-stage renal disease requiring maintenance hemodialysis; (6) Active chemotherapy or radiotherapy for malignancy.

Review and collect clinical data of patients with acute STEMI, including past disease history, family history, medication history, smoking history, and alcohol consumption history. The basic data of patients at the time of emergency reception, including gender, age, complications, risk factors (such as smoking, drinking, diabetes, hypertension, family history of cardiovascular and cerebrovascular diseases), cardiac and non-cardiac symptoms (such as chest pain, dyspnea, diaphoresis, vomiting, fatigue, etc.), onset to hospital time, pre hospital vital signs (blood pressure, heart rate), and emergency ECG parameters (heart rate, *P*-wave duration, PR interval, QRS duration, QT/QTc, U-wave, arrhythmia, infarction site, etc.). Arrhythmias include ventricular arrhythmias (such as premature ventricular contractions, transient ventricular tachycardia, etc.) and atrial arrhythmias (such as premature atrial contractions, atrial tachycardia etc.). All these data were collected within 1 h after the patient arrived at the emergency department. All indicators can be synchronously obtained in the prehospital first-aid process through on-site inquiry and prehospital 12-lead electrocardiogram.

All patients completed venous potassium collection within 1 h of arrival at the emergency room, and concentrations were measured by direct ion-selective electrode with a reference interval of 3.5–5.1 mmol/L. Except the serum potassium required for the diagnosis of hypokalemia, it does not include any other laboratory examination items.

This study divided patients into two groups based on their blood potassium levels. One group was the hypokalemia group (Blood potassium level is less than 3.5 mmol/L), with a total of 114 cases, including 101 males and 13 females; the other group was the non-hypokalemia group (Blood potassium level is above than 3.5 mmol/L), with a total of 206 cases, including 172 males and 34 females.

## Statistical analysis

Statistical analyses were performed using SPSS version 27.0 and R software (version 4.2.2). Normality was assessed using the Shapiro–Wilk test. Normally distributed variables are presented as mean ± standard deviation, Independent sample *t*-test is used for inter group comparison. Non-normally distributed variables were expressed as median (interquartile range), with between-group comparisons performed using the Wilcoxon rank-sum test. Categorical variables are presented as counts and percentages (n/%), with between-group comparisons conducted using *χ*² test or Fisher's exact test as appropriate. Screening independent influencing factors of hypokalemia through univariate logistic regression, least absolute shrinkage and selection operator (LASSO), and multivariate logistic regression. Construct a diagnostic prediction model for prehospital hypokalemia in STEMI patients based on the independent influencing factors obtained and visualize it in a nomogram. Evaluate the predictive model from three aspects: discrimination, calibration, and clinical effectiveness. The discriminability is evaluated by plotting the ROC curve and calculating the area under the curve (AUC), the model calibration is validated by the calibration curve, and the clinical effectiveness of the model is evaluated using the clinical decision curve (DCA). Using Bootstrap method for internal validation of the model to obtain corrected AUC.

## Results

### Baseline characteristics

Baseline characteristics revealed no significant differences in age, gender, BMI, or medical history between hypokalemic and non-hypokalemic patients. The distribution of infarct-related coronary territories did not differ significantly between the hypokalemic and non-hypokalemic cohorts. Clinically, hypokalemic patients presented with shorter symptom-to-door time [median 2.0 (IQR 1.0–3.0) vs. 2.0 (IQR 1.0–5.0) hours, *p* < 0.001], lower admission systolic blood pressure (134 ± 30 vs. 142 ± 28 mmHg, *p* = 0.025), and higher rates of diaphoresis and syncope/coma. Electrocardiographic analysis revealed significantly longer PR intervals in hypokalemic patients [175 (IQR 155–188) vs. 165 (IQR 151–180) ms, *p* = 0.006], with higher prevalence of atrial arrhythmias and U-wave presence (Table 1).

**Table 1:** Comparisons of characteristics between hypokalemia and non-hypokalemia.

Variables	Overall	Non-hypokalemia group	Hypokalemia group	Statistic	*p* Value
	*N* = 320	*N* = 206	*N* = 114		
Potassium	3.69 (3.40, 3.98)	3.90 (3.72, 4.13)	3.32 (3.16, 3.41)	23,484.	<0.001
Basic information					
Age(years)	58 (48, 68)	58 (44, 69)	58 (52, 65)	11,627	0.885
BMI, kg/m²	25.1 (22.5, 27.1)	24.9 (22.2, 27.4)	25.1 (22.8, 27.0)	10,577	0.878
Male, n(%)	273 (85.3%)	172 (83.5%)	101 (88.6%)	1.52	0.217
Smoking	183 (57.2%)	117 (56.8%)	66 (57.9%)	0.04	0.849
Medical and medication history					
Hypertension	201 (62.8%)	132 (64.1%)	69 (60.5%)	0.4	0.529
Diabetes mellitus	70 (21.9%)	49 (23.8%)	21 (18.4%)	1.24	0.266
Prior MI	21 (6.6%)	16 (7.8%)	5 (4.4%)	1.37	0.242
Cerebrovascular disease	21 (6.6%)	15 (7.3%)	6 (5.3%)	0.49	0.485
ACEI/ARB	60 (18.8%)	42 (20.4%)	18 (15.8%)	1.02	0.313
CCB	78 (24.4%)	56 (27.2%)	22 (19.3%)	2.48	0.116
Beta-blocker	33 (10.3%)	21 (10.2%)	12 (10.5%)	0.01	0.925
Insulin	14 (4.4%)	8 (3.9%)	6 (5.3%)	0.33	0.577
Metformin	27 (8.4%)	18 (8.7%)	9 (7.9%)	0.07	0.795
Diuretics	15 (4.7%)	9 (4.4%)	6 (5.3%)	0.13	0.717
Disease situation					
Symptom-to-door time, h	2.0 (1.0, 4.0)	2.0 (1.0, 5.0)	2.0 (1.0, 3.0)	14,582.	<0.001
SBP, mmHg	139 ± 29	142 ± 28	134 ± 30	2.26	0.025
DBP, mmHg	87 (72, 101)	88 (74, 101)	85 (71, 100)	12,647	0.253
Vomiting	67 (20.9%)	47 (22.8%)	20 (17.5%)	1.23	0.267
Diaphoresis	114 (35.6%)	82 (39.8%)	32 (28.1%)	4.41	0.036
Syncope/Coma	15 (4.7%)	6 (2.9%)	9 (7.9%)	4.08	0.043
Killip classification				0.63	0.889
1	193 (60.3%)	124 (60.2%)	69 (60.5%)		
2	65 (20.3%)	44 (21.4%)	21 (18.4%)		
3	17 (5.3%)	10 (4.9%)	7 (6.1%)		
4	45 (14.1%)	28 (13.6%)	17 (14.9%)		
Electrocardiogram parameters					
Heart rate, bpm	75 (65, 87)	75 (65, 85)	74 (62, 91)	11,705.	0.964
*P*-wave duration, ms	93 (87, 102)	93 (86, 100)	95 (89, 105)	10,157.	0.046
PR-interval, ms	169 (152, 183)	165 (151, 180)	175 (155, 188)	9,582.5	0.006
QRS duration, ms	98 (90, 104)	97 (91, 103)	98 (90, 104)	11,465.	0.727
QT interval, ms	367 (344, 395)	367 (345, 393)	368 (343, 401)	11,584.	0.842
QTc interval, ms	409 (392, 430)	408 (390, 430)	410 (395, 431)	11,108.	0.424
Atrial arrhythmia	16 (5.0%)	5 (2.4%)	11 (9.6%)	8.06	0.005
Ventricular arrhythmia	15 (4.7%)	13 (6.3%)	2 (1.8%)	3.41	0.065
U wave present	47 (14.7%)	16 (7.8%)	31 (27.2%)	22.1	<0.001
Myocardial infarction site					0.536
Extensive anterior wall	63 (19.7%)	37 (18.0%)	26 (22.8%)		
Anterior wall	122 (38.1%)	78 (37.9%)	44 (38.6%)		
Lateral wall	121 (37.8%)	80 (38.8%)	41 (36.0%)		
Posterior wall	14 (4.4%)	11 (5.3%)	3 (2.6%)		

Continuous data are presented as mean ± SD or median (Q1, Q3); categorical data are presented as count (%). *P* values are derived from *t* tests for continuous variables and chi-square tests for categorical variables.

BMI, body mass index; ACEI/ARB, angiotensin-converting enzyme inhibitor or angiotensin receptor blocker; CCB, calcium channel blocker; SBP, systolic blood pressure; DBP, diastolic blood pressure.

### Logistic regression and LASSO regression

**Figure 1:**
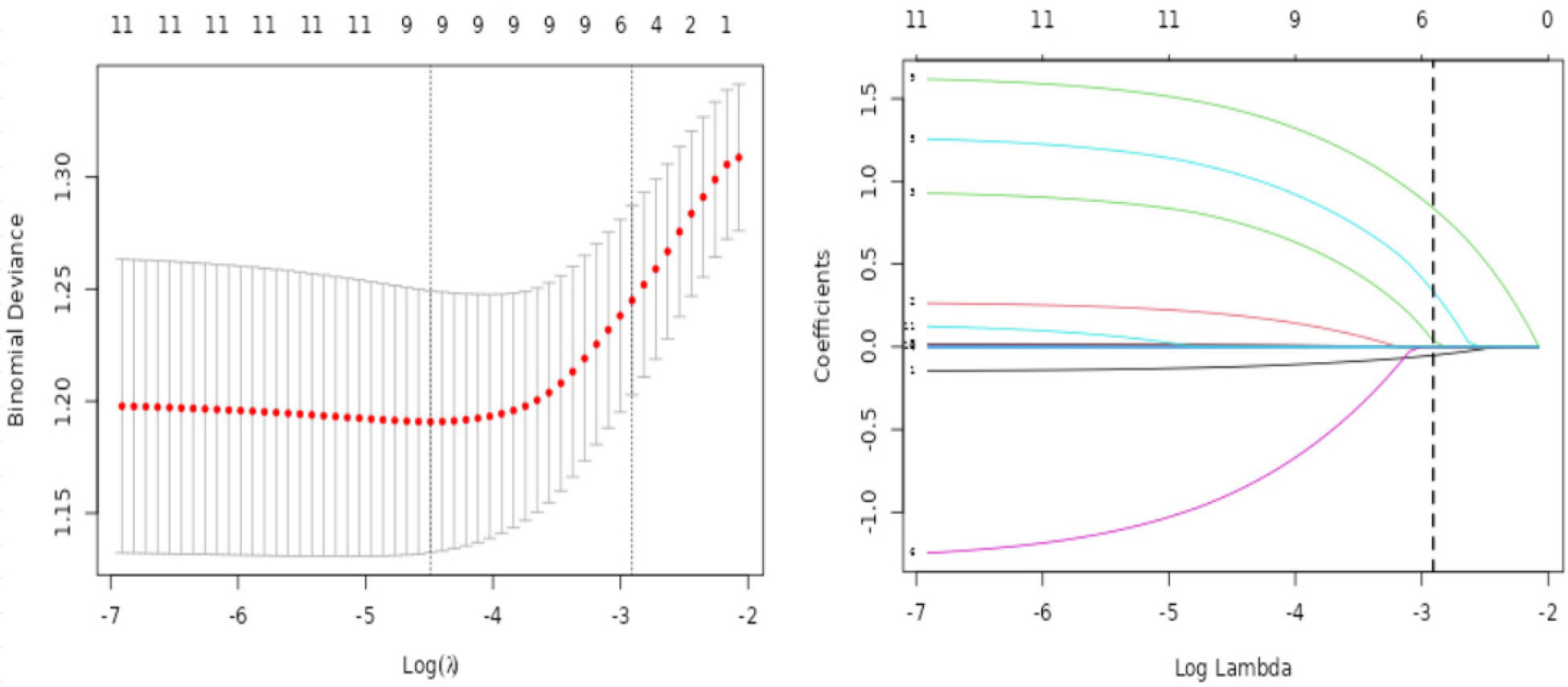
The least absolute contraction and selection operator (LASSO) logistic regression is used for feature selection. On the left, the LASSO coefficient path diagram of 11 influencing factors is shown, and on the right, the 10-fold cross-validation curve is shown.

Univariate analysis identified symptom-to-door time, diaphoresis, syncope/coma, and admission systolic blood pressure as potential predictors of hypokalemia. Electrocardiographic findings including atrial arrhythmias, ventricular arrhythmias, U-wave presence, prolonged *P*-wave duration, and extended PR interval emerged as potential risk factors (Table 2). Incorporate basic demographic factors (age, gender) and select variables with *p* values less than 0.10 from single factors: Symptom-to-door time, diaphoresis, syncope/coma, systolic blood pressure, atrial arrhythmia, ventricular arrhythmia, U-wave presence, *P*-wave duration, and PR interval. Using 10-fold cross-validation (nlambda = 100), LASSO regression identified six variables with non-zero coefficients at the optimal penalty parameter (*λ*.1se = 0.054): Symptom-to-door time, systolic blood pressure, syncope/coma, ventricular arrhythmia, PR interval, and U-wave presence (Figure 1). The six LASSO-selected variables were incorporated into a multivariate logistic regression model with hypokalemia as the outcome variable. Variable selection was performed using backward stepwise regression, with *α* = 0.05 as the exclusion threshold. The final model comprised five independent predictors: shorter symptom-to-door time (OR = 0.85), syncope/coma (OR = 3.57), atrial arrhythmias (OR = 4.18), prolonged PR interval (OR = 1.01), and U-wave presence (OR = 5.20) (Table 3).

**Table 2:** Univariate logistic regression analysis for predictors of hypokalemia.

Variables	Event *N*	OR	95% CI	*p*-value
Symptom-to-door time	114	0.86	0.79, 0.94	0.001
Diaphoresis	82	1.69	1.03, 2.78	0.037
Syncope/Coma	9	2.86	0.99, 8.24	0.052
SBP	114	0.99	0.98, 1.00	0.023
*P*-wave duration	114	1.02	1.00, 1.05	0.044
PR interval	114	1.01	1.00, 1.02	0.009
U wave present	31	4.44	2.30, 8.55	<0.001
Atrial arrhythmia	11	4.29	1.45, 12.69	0.008
Ventricular arrhythmia	2	0.27	0.06, 1.20	0.084

Analyses using logistic regression models.

OR, odds ratio; 95% CI, 95% confidence interval; SBP, systolic blood pressure.

**Table 3:** Multivariate logistic regression analysis of hypokalemia in STEMI patients.

Characteristic	*N*	Event *N*	OR	95% CI	*p*-value
Symptom-to-door time	320	114	0.85	0.78, 0.94	0.002
Syncope/Coma	15	9	3.57	1.12, 11.37	0.031
Atrial arrhythmia	16	11	4.18	1.33, 13.17	0.015
PR interval	320	114	1.01	1.00, 1.02	0.019
U wave present	47	31	5.2	2.59, 10.46	<0.001

Analyses using logistic regression models.

OR, odds ratio; 95% CI, 95% confidence interval.

### Establishment of nomogram

**Figure 2:**
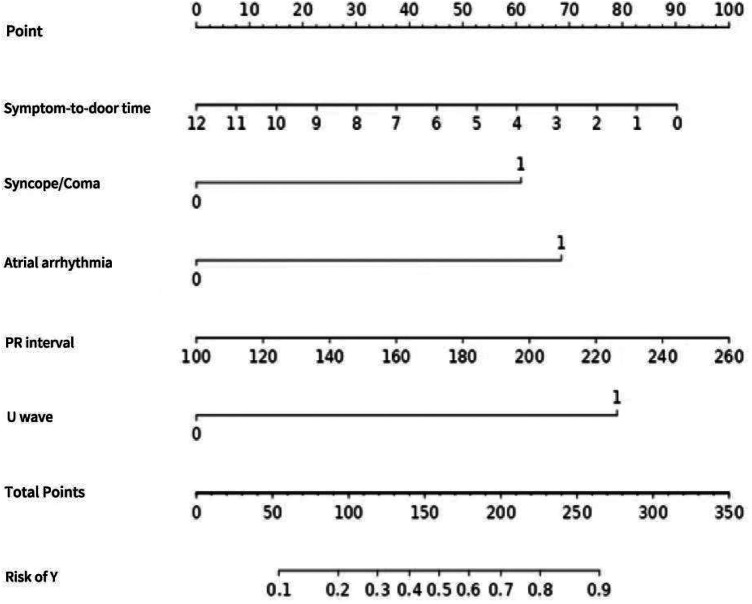
Nomogram of multi-factor joint prediction model for hypokalemia in STEMI patients.

Based on 5 influencing factors (symptom-to-door time, syncope/coma, atrial arrhythmia, PR interval, U-wave) selected by multiple logistic regression were entered into the joint prediction model. A nomogram is constructed, showing the values of each independent influencing factor and the corresponding scores of each influencing factor. The scores of each influencing factor are added up to obtain the total score, and its corresponding risk level is the probability of predicting the occurrence of hypokalemia (Figure 2).

### Prediction model evaluation

**Figure 3:**
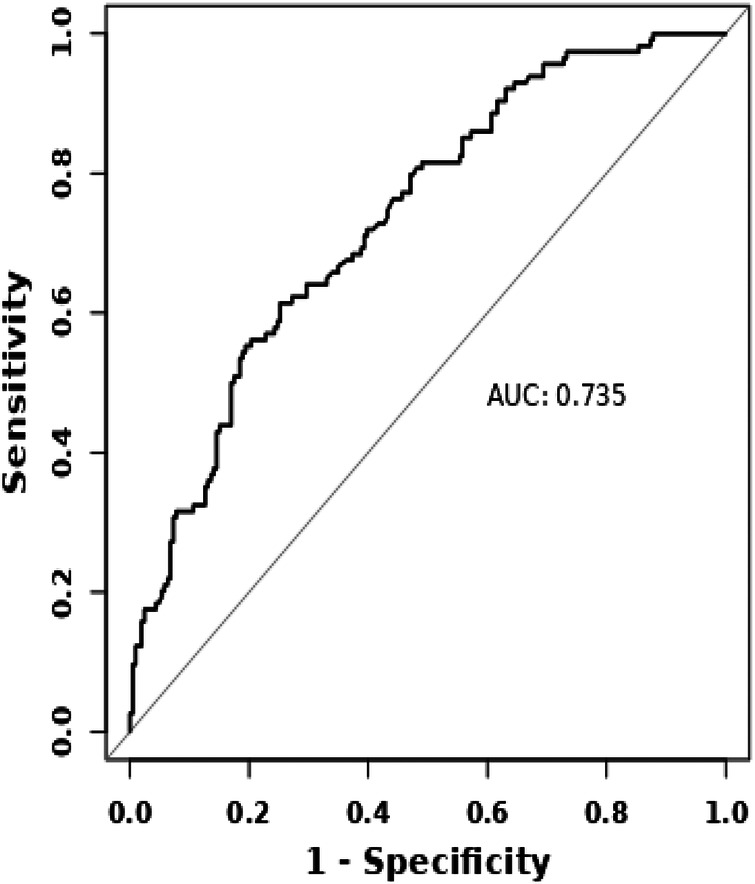
ROC curve of prediction model of hypokalemia in STEMI patients.

**Figure 4:**
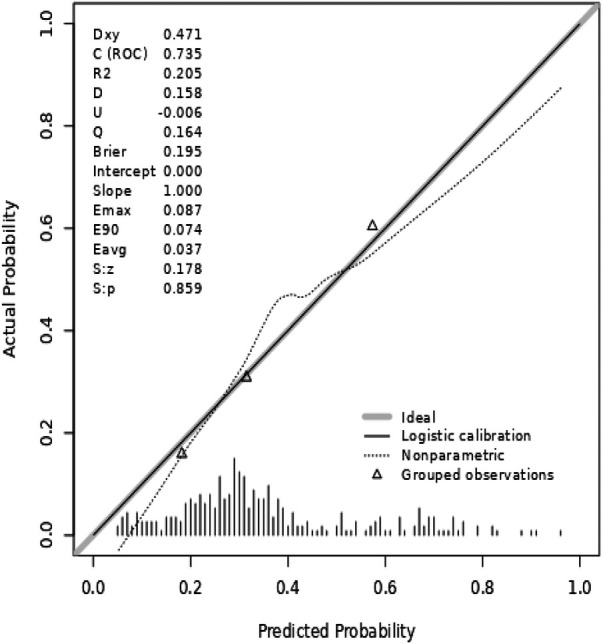
Calibration curve of multivariate joint prediction model for hypokalemia in STEMI patients.

**Figure 5:**
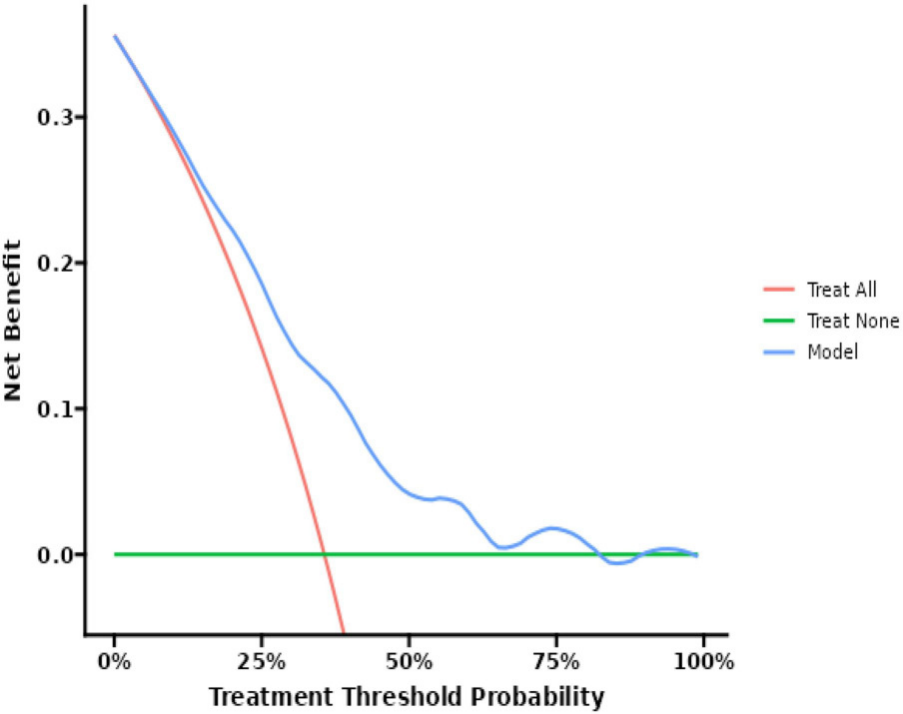
DCA curve of multi-factor joint prediction model for hypokalemia in STEMI patients.

Perform ROC analysis on the joint prediction model, and the AUC was 0.735 with a 95% confidence interval of 0.680–0.791 (Figure 3). Internal validation with 1,000 bootstrap resamples demonstrated robust model performance, yielding a corrected C-statistic of 0.700. This indicates preferable discrimination and calibration. The calibration curve (Figure 4) indicates that there is good consistency between the model prediction and the actual observation results, proving the superiority of the fitted model. The DCA curve results indicate that when the threshold probability is within the range of 10% to 80%, applying the model to guide clinical decision-making within this range has good clinical benefits (Figure 5).

## Discussion

This study focuses on pre-hospital emergency scenarios and constructs and validates a hypokalemia risk prediction model practical, concise variables, and good predictive performance. Using LASSO and multivariate logistic regression, we identified five key predictors of prehospital hypokalemia: symptom-to-door time, syncope/coma, atrial arrhythmias, PR interval prolongation, and U-wave presence. The significance of these predictive factors lies in their ability to provide early signals of hypokalemia. This is easily overlooked in acute STEMI attacks. For example, the Symptom-to-door time and the presence of syncope or coma reflect the urgency and potential severity of the patient's condition. Atrial arrhythmia, prolonged PR interval, and U-wave are signs of electrophysiological disorders in patients. Based on these factors, we constructed a nomogram. Our prediction model achieved an AUC of 0.735 (95% CI: 0.680–0.791) on ROC analysis, indicating reasonably strong discriminative ability. Notably, manifestations such as muscle weakness, U-wave appearance, and arrhythmias are common to both hypokalemia and acute myocardial infarction, complicating early differentiation (8). The prediction model, built upon five convenient and easily accessible variables, demonstrated robust performance. It should be clearly clarified that the data used for model construction were collected from the emergency department, while all 5 predictors included in the final model can be quickly obtained in the prehospital setting without invasive laboratory testing. Therefore, the core advantage of this model is that it can be completely translated and applied to the prehospital emergency workflow to realize early risk stratification of hypokalemia before hospital admission. This model can assist medical staff in identifying patients at high risk of hypokalemia in a convenient and efficient manner during busy and tense emergency work. On the one hand, it enhances medical staff's attention to patients' hypokalemia risks, enabling them to prioritize the initiation of urgent serum potassium testing procedures. On the other hand, it provides evidence-based references for clinical decision-making regarding early preventive potassium supplementation, truly achieving early identification and intervention of hypokalemia risks and reducing the occurrence risk of hypokalemia-related malignant arrhythmias.

Previous retrospective studies consistently demonstrate that STEMI patients frequently develop hypokalemia within 12 h of onset, with associated increases in malignant arrhythmias and in-hospital mortality (9, 10). This study also found that early onset hypokalemia can occur in STEMI patients. This phenomenon likely reflects acute stress responses characterized by sympathetic nervous system activation, massive catecholamine release, and β2-receptor-mediated potassium shifts into cells. Specifically designed for early hypokalemia detection in STEMI, the model's clinical value was quantified by decision curve analysis, which indicated significant net benefits over a wide spectrum of threshold probabilities. This suggests its potential to guide clinicians in making superior decisions regarding potassium supplementation, with the ultimate goal of lowering arrhythmia-related mortality and enhancing patient outcomes.

Syncope is one of the important atypical manifestations of STEMI patients, and some STEMI patients present with syncope as the initial symptom rather than atypical chest pain, which can be easily misdiagnosed or delayed in treatment (11). In our cohort, syncope/coma emerged as a robust predictor of hypokalemia (OR = 3.57). STEMI itself can cause abnormal blood potassium levels through the release of potassium from necrotic myocardium, stress response, and impaired renal function, which can directly lead to fatal arrhythmias, resulting in syncope/coma. In addition, low potassium itself can also cause a decrease in neuromuscular excitability, which can manifest as muscle weakness, respiratory depression, and even consciousness disorders. Pre-hospital identification of such symptoms should be highly alert to the occurrence of electrolyte imbalances.

Atrial arrhythmias (premature atrial contractions and transient atrial tachycardia) represent both important clinical manifestations of hypokalemia and independent predictors in our analysis (OR = 4.18). Hypokalemia can significantly prolong the duration of atrial action potential by inhibiting fast delayed rectifier potassium current (IKr) and fast delayed rectifier potassium current (IKur) on the atrial muscle cell membrane, and ultimately triggering atrial arrhythmia (12). The pig acute myocardial infarction model constructed by Bikou et al. showed that within 2 h after myocardial infarction, after using some mechanical devices to replace left ventricular function, and the incidence of atrial arrhythmias also decreased, confirming that “mechanical electrical feedback” is a reversible arrhythmogenic factor (13). At the clinical level, although the incidence of atrial arrhythmia in STEMI patients is lower than that of ventricular arrhythmia, its value as an early signal of hypokalemia cannot be ignored. A study based on the risk of atherosclerosis in the community found that the incidence of atrial arrhythmias events in the population with coronary heart disease and hypokalemia was as high as 19.84% (14). We hypothesize a self-perpetuating cycle in STEMI: ischemic-driven catecholamine release lowers serum potassium, which facilitates atrial arrhythmias through altered depolarization/repolarization. These arrhythmias, compounded by ischemia-induced atrial stretch, further impair hemodynamics and coronary perfusion, worsening ischemia and perpetuating hypokalemia. This electromechanical vicious cycle underscores that new atrial arrhythmias warrant prompt potassium evaluation and correction.

The prolongation of PR interval reflects the delay of atrioventricular node conduction. Hypokalemia delays atrioventricular conduction through multiple mechanisms, including sodium-potassium pump inhibition and reduced resting membrane potential. A case report of severe hypokalemia (1.31 mmol/L) confirmed that the prolongation of PR interval after potassium supplementation is reversible (15). Therefore, detecting prolonged PR interval in STEMI patients can serve as a warning signal for hypokalemia, and the recovery of PR interval after potassium supplementation can also serve as an effective indicator of potassium supplementation. It has strong operability and repeatability in pre-hospital emergency care. Zareei et al. found in 248 patients with acute coronary syndrome that PR interval prolongation can serve as a potential marker of cardiac structure/ischemic load (16). However, further research is needed to determine whether the PR interval can directly reflect ischemia in the atrioventricular node of STEMI patients.

U-waves represent a hallmark electrocardiographic marker of hypokalemia. When hypokalemia occurs, the outward potassium current (such as IKr, Ito) of myocardial cells weakens, resulting in asynchronous repolarization between ventricular myocardium and conduction system, thus forming U-waves (17). Ramadurai et al. found that the incidence of U-waves significantly increased with the severity of hypokalemia (18). It is worth noting that the clinical significance of U-waves seems to be not limited to electrolyte imbalance. Inverted U-waves may represent an early, non-invasive marker of acute myocardial ischemia, even in the absence of canonical ST-T changes, as evidenced by a case study from Girish et al. (19). In our study, U-waves were confirmed to be an independent predictor of prehospital hypokalemia in STEMI patients (OR = 5.2, 95% CI: 2.59–10.46, *p* < 0.001). The appearance of U-waves may not only reflect repolarization abnormalities caused by low potassium, but may also be combined with myocardial electrophysiological disorders caused by ischemia. However, U-waves can also occur in some healthy individuals such as athletes and those with bradycardia, so the quantitative relationship between U-wave morphology (positive/negative, amplitude, duration) and blood potassium levels, as well as the degree of coronary artery disease, still needs further exploration in the future.

A recently published large-scale retrospective cohort study based on the MIMIC-IV database found a significant positive correlation between serum potassium fluctuations and in-hospital mortality (20). The study by Fax é n et al. demonstrated a positive correlation between hypokalemia upon admission and adverse events during hospitalization (21). Petnak et al. found that low blood potassium at discharge is significantly associated with one-year mortality (22). These pieces of evidence all indicate that hypokalemia is an independent risk factor for death in STEMI patients, and its electrophysiological mechanism may be that after myocardial infarction occurs, the expression and function of potassium channels in myocardial cells undergo remodeling downregulation, leading to prolonged action potential duration and increased repolarization dispersion. This electrical remodeling itself puts the myocardium in a vulnerable state, and the presence of hypokalemia can further inhibit potassium channel function, weaken the ischemic preconditioning protective effect mediated by potassium ion channels, aggravate calcium influx and intracellular calcium overload, thereby inducing or exacerbating arrhythmia and increasing mortality (23).

Although the significance of in-hospital potassium management has been well documented, far less attention has been paid to the early, prehospital detection of hypokalemia. Our study bridges this gap by integrating five straightforward clinical and electrocardiographic parameters into a nomogram. This approach facilitates rapid risk assessment at the bedside, significantly enhancing its utility in emergent prehospital settings.

## Study limitations

Although this study represents a positive exploration in model construction and validation, several limitations must be acknowledged. First, as a single-center retrospective study conducted in a tertiary hospital in eastern China, the enrolled patients were predominantly from the local Han population, and the region benefits from a well-established prehospital care system and referral network. These factors inherently limit the generalizability of our findings to other geographical regions, different tiers of healthcare institutions, and diverse ethnic groups. Selection and information biases cannot be entirely ruled out. Therefore, future multicenter prospective cohort studies across varied regions and healthcare systems are warranted to validate the stability and transportability of the model. Second, our analysis focused solely on admission potassium levels, which restricts insights into the dynamic changes in serum potassium over time. Incorporating continuous potassium monitoring through time-to-event models (e.g., Cox regression, landmark analysis) in future investigations would better characterize the temporal patterns of hypokalemia development. Third, while the model demonstrates potential for early risk stratification, its clinical impact remains to be proven. Future randomized controlled trials should be designed to evaluate whether a model-guided preventive potassium supplementation strategy can reduce the incidence of arrhythmias and improve mortality, thereby closing the loop from prediction to intervention.

## Conclusion

We developed and validated a practical nomogram for predicting hypokalemia in STEMI patients using five variables readily available in prehospital and emergency settings. All predictors can be obtained immediately through history taking and the initial 12-lead ECG, without waiting for laboratory results. This tool enables rapid risk stratification, facilitates early identification of high-risk individuals, and supports timely decision-making for preventive potassium supplementation, thereby helping to reduce the risk of hypokalemia-related malignant arrhythmias and improve clinical outcomes.

## Data Availability

The raw data supporting the conclusions of this article will be made available by the authors, without undue reservation.

## References

[1] Weiss JN Qu Z Shivkumar K. Electrophysiology of hypokalemia and hyperkalemia. Circ Arrhythm Electrophysiol. (2017) 10:e004667. 10.1161/CIRCEP.116.004667 28314851 PMC5399982

[2] Wang Y Li Q Tao B Angelini M Ramadoss S Sun B Fibroblasts in heart scar tissue directly regulate cardiac excitability and arrhythmogenesis. Science. (2023) 381:1480–7. 10.1126/science.adh9925 37769108 PMC10768850

[3] Colombo MG Kirchberger I Amann U Dinser L Meisinger C. Association of serum potassium concentration with mortality and ventricular arrhythmias in patients with acute myocardial infarction: a systematic review and meta-analysis. Eur J Prev Cardiol. (2018) 25:576–95. 10.1177/2047487318759694 29473462

[4] Xi H Yu R-H Wang N Chen X-Z Zhang W-C Hong T. Serum potassium levels and mortality of patients with acute myocardial infarction: a systematic review and meta-analysis of cohort studies. Eur J Prev Cardiol. (2019) 26:145–56. 10.1177/2047487318780466 31060369

[5] Ravn Jacobsen M Jabbari R Glinge C Kjær Stampe N Butt JH Blanche P Potassium disturbances and risk of ventricular fibrillation among patients with ST-segment–elevation myocardial infarction. J Am Heart Assoc. (2020) 9:e014160. 10.1161/JAHA.119.014160 32067598 PMC7070188

[6] Lawton JS Tamis-Holland JE Bangalore S Bates ER Beckie TM Bischoff JM 2021 ACC/AHA/SCAI guideline for coronary artery revascularization: a report of the American College of Cardiology/American Heart Association joint committee on clinical practice guidelines. Circulation. (2022) 145:e21–e129. 10.1161/CIR.0000000000001038 34882435

[7] Thygesen K Alpert JS Jaffe AS Chaitman BR Bax JJ Morrow DA Fourth universal definition of myocardial infarction (2018). Circulation. (2018) 138:e618–51. 10.1161/CIR.0000000000000617 30571511

[8] Kardalas E Paschou SA Anagnostis P Muscogiuri G Siasos G Vryonidou A. Hypokalemia: a clinical update. Endocr Connect. (2018) 7:R135–46. 10.1530/EC-18-0109 29540487 PMC5881435

[9] Jensen CJ Nielsen JK Talbott MM O’Connell D Patel VS Armstrong PA Effects of serum potassium on mortality in patients with ST-elevation myocardial infarction. Cureus. (2024) 16:e61126. 10.7759/cureus.61126 38919213 PMC11197046

[10] Uluganyan M Ekmekçi A Murat A Avşar Ş Ulutaş TK Uyarel H Admission serum potassium level is associated with in-hospital and long-term mortality in ST-elevation myocardial infarction. Anatol J Cardiol. (2016) 16(1):10–5. 10.5152/akd.2015.5706 26467357 PMC5336698

[11] Sato N Minami Y Ako J Maeda A Akashi Y Ikari Y Clinical significance of prehospital 12-lead electrocardiography in patients with ST-segment elevation myocardial infarction presenting with syncope: from a multicenter observational registry (K-ACTIVE study). Heart Vessels. (2021) 36:1466–73. 10.1007/s00380-021-01832-z 33710375

[12] Tazmini K Frisk M Lewalle A Laasmaa M Morotti S Lipsett DB Hypokalemia promotes arrhythmia by distinct mechanisms in atrial and ventricular myocytes. Circ Res. (2020) 126:889–906. 10.1161/CIRCRESAHA.119.315641 32070187 PMC7098435

[13] Bikou O Kho C Ishikawa K. Atrial stretch and arrhythmia after myocardial infarction. Aging. (2018) 11:11–2. 10.18632/aging.101729 30594913 PMC6339802

[14] Wu Y Kong X-J Ji Y-Y Fan J Ji C-C Chen X-M Serum electrolyte concentrations and risk of atrial fibrillation: an observational and Mendelian randomization study. BMC Genomics. (2024) 25:280. 10.1186/s12864-024-10197-2 38493091 PMC10944597

[15] Wang X Han D Li G. Electrocardiographic manifestations in severe hypokalemia. J Int Med Res. (2020) 48:0300060518811058. 10.1177/0300060518811058 30509119 PMC7287199

[16] Zareei M Zareiamand H Kamali M Ardalani N Ebrahimi A Nabati M. Can prolonged P-R interval predict clinical outcomes in non-ST elevation acute coronary syndrome patients? BMC Cardiovasc Disord. (2024) 24:137. 10.1186/s12872-024-03809-y 38431589 PMC10909255

[17] Kihlgren M Almqvist C Amankhani F Jonasson L Norman C Perez M The U-wave: a remaining enigma of the electrocardiogram. J Electrocardiol. (2023) 79:13–20. 10.1016/j.jelectrocard.2023.03.001 36907158

[18] Ramadurai S Varadarajan V Varadarajan S Venkatasubramanian K. Electrocardiographic changes in patients with hypokalemia and their correlation with serum potassium levels. Cureus. (2025) 17:e91922. 10.7759/cureus.91922 41080383 PMC12510615

[19] Mp G Gupta MD Mukhopadhyay S Yusuf J Tn SR. U wave: an important noninvasive electrocardiographic diagnostic marker. Indian Pacing Electrophysiol J. (2005) 5(1):63–5. 16943944 PMC1502069

[20] Zhou Y Chen Y Liang S Li Y Zhao C Wu Z. Association between potassium fluctuation and in-hospital mortality in acute myocardial infarction patients: a retrospective analysis of the MIMIC-IV database. Clin Res Cardiol. (2025). 10.1007/s00392-025-02613-8 39939529

[21] Faxén J Xu H Evans M Jernberg T Szummer K Carrero J-J. Potassium levels and risk of in-hospital arrhythmias and mortality in patients admitted with suspected acute coronary syndrome. Int J Cardiol. (2019) 274:52–8. 10.1016/j.ijcard.2018.09.099 30282599

[22] Thongprayoon C Cheungpasitporn W Thirunavukkarasu S Petnak T Chewcharat A Bathini T Serum potassium levels at hospital discharge and one-year mortality among hospitalized patients. Medicina (Mex). (2020) 56:236. 10.3390/medicina56050236 PMC7279137 32423140

[23] Song T Hui W Huang M Guo Y Yu M Yang X Dynamic changes in ion channels during myocardial infarction and therapeutic challenges. Int J Mol Sci. (2024) 25:6467. 10.3390/ijms25126467 38928173 PMC11203447

